# *Salmonella* Typhi Shedding and Household Transmission by Children With Blood Culture-Confirmed Typhoid Fever in Vellore, South India

**DOI:** 10.1093/infdis/jiab409

**Published:** 2021-11-23

**Authors:** Manikandan Srinivasan, Kulandaipalayam Natarajan Sindhu, Sidhartha Giri, Nirmal Kumar, Venkata Raghava Mohan, Nicholas C Grassly, Gagandeep Kang

**Affiliations:** 1 The Wellcome Trust Research Laboratory, Division of Gastrointestinal Sciences, Christian Medical College, Vellore,India; 2 Indian Council of Medical Research – Regional Medical Research Centre, Bhubaneswar, Odisha,India; 3 Department of Community Health, Christian Medical College, Vellore,India; 4 Department of Infectious Disease Epidemiology, Imperial College, London,United Kingdom

**Keywords:** children, India, *Salmonella* Typhi, shedding, typhoid fever

## Abstract

**Background:**

Children suffer the highest burden of the typhoid fever, with a considerable proportion shedding *Salmonella* Typhi in stool, potentially resulting in transmission of *S* Typhi.

**Methods:**

We enrolled 70 children with blood culture-confirmed typhoid fever (index cases), from 63 households, during community-based fever surveillance in India. The index cases and their household contacts were followed up with stool samples at multiple time points over 3 weeks and 1 week, respectively. *S* Typhi was detected using quantitative real-time polymerase chain reaction.

**Results:**

Fifteen of 70 (21.4%) children with culture-confirmed typhoid fever shed *S* Typhi in stool after onset of fever. Ten of 15 children shed *S* Typhi for a median of 11.5 (range, 3–61) days from the day of completion of antibiotics. Of 172 household contacts from 56 of the 63 index case households, 12 (7%) contacts in 11 (19.6%) households had *S* Typhi in stool. Five of the 12 contacts who were shedding *S* Typhi were asymptomatic, whereas 7 reported recent fever.

**Conclusions:**

One in 5 children with typhoid fever shed *S* Typhi, with shedding persisting even after antibiotics. One in 5 households had at least 1 contact of the child shedding *S* Typhi, highlighting potential concurrent typhoid infections in households in settings with poor water and sanitation.

*Salmonella enterica* serovar Typhi (*Salmonella* Typhi), a human host-restricted facultative bacillus, accounts for 11 million cases of typhoid fever annually with an estimated 116 815 typhoid-related deaths worldwide [1]. India, where typhoid is endemic, has an estimated incidence of 377 (178–801) cases of typhoid fever per 100 000 person-years [2]. The Surveillance for Enteric Fever in India (SEFI) study has estimated an incidence of ~1000 cases of typhoid fever in children <15 years per 100 000 person-years in urban Vellore (submitted for publication). The transmission of *Salmonella* Typhi is predominantly fecal-oral, particularly occurring in the urban or periurban communities of developing countries, where there is frequent contamination of drinking water with human fecal bacteria [3, 4]. Spread of *S* Typhi within communities can occur through a combination of short-cycle (contamination of food and water in the immediate vicinity of the case) and long-cycle (contamination of water supply systems within the wider community) transmission [5]. Young children in low-income settings, given lack of pre-existing immunity from prior infection to *S* Typhi and high exposure to the pathogen, are thought to contribute greatly to transmission [6].

*Salmonella* Typhi can persist, but with a limited capacity to multiply, in the external environment [5, 7, 8]. An individual, once infected, can shed *S* Typhi in feces and occasionally in urine during the incubation period, or during the acute or convalescent phase after an episode of typhoid fever (temporary shedding) [5]. Shedding of *S* Typhi can also occur with subclinical infections and as a consequence of chronic carriage in a small subset of individuals (chronic carriers) [9]. Approximately 10% of individuals with untreated typhoid shed *S* Typhi during convalescence, and ~1–4% of these individuals continue to shed even after a year [10]. These individuals shedding *S* Typhi, as temporary carriers, are important drivers of disease transmission within the community, especially in endemic settings [5].

After entry of *S* Typhi into the host, there is rapid multiplication in the gut, with shedding of *S* Typhi within the first 24 hours after infection [11]. This shedding of *S* Typhi is intermittent [10]. Human challenge studies have demonstrated that more than half of the subjects infected with *S* Typhi shed the bacilli at least once within the first 14 days after the challenge, demonstrating patterns of early- and late-phase shedding [12]. The bulk of shedding (38%) was evident by day 1 and 2 post-challenge (primary peak), and this conforms with the incubation period after natural infection with *S* Typhi. The primary peak was followed by a steady decrease in shedding until the later part of the second week when a smaller secondary peak was observed. Shedding was also seen in a small proportion (9%) of asymptomatic subjects who shed bacilli up to 3 weeks after the challenge, showing the same bimodal peak as their symptomatic counterparts. The challenged subjects who subsequently developed clinical typhoid fever had a 25% higher risk of shedding *S* Typhi in the first 3 days after the challenge compared with those who were asymptomatic [12].

The dosage and duration of antimicrobial therapy is an important determinant of *S* Typhi shedding during acute infection, as shown in a human challenge study [12]. Shedding waxed and waned with temporary cessation and resumption of antibiotics during treatment in the challenged subjects. Those treated with chloramphenicol/amoxicillin/co-trimoxazole had higher stool culture positivity (3.5%–6%) compared with those on azithromycin/cefixime/ceftriaxone (2%) [10]. Human challenge studies have offered a comprehensive insight into the shedding dynamics of *S* Typhi. However, these studies have primarily been conducted in adults in developed settings where subjects are immunologically naive for typhoid, in contrast to endemic developing countries, where children are more likely to be infected.

Examining and understanding the pattern of shedding of *S* Typhi, especially in children who constitute the bulk of the disease burden in low- and middle-income settings is essential to influence strategies to decrease disease transmission and reduce the overall burden [13]. Our study was nested within the SEFI cohort of ~6000 children aged between 0.5 and 15 years at Vellore. We aimed to estimate the proportion of *S* Typhi shedding among children with blood culture-confirmed cases of typhoid fever and screen their household contacts to study household-level transmission dynamics.

## METHODS

### Study Setting and Participants

The SEFI study was carried out between October 2017 and December 2019 at 4 sites: Delhi, Kolkata, Pune, and Vellore, to estimate the incidence rate of blood culture-confirmed typhoid fever in children aged between 6 months and 15 years as per a published protocol [14]. At each site, a cohort of ~6000 children was followed up weekly to capture fevers. Fevers for ≥3 days, with the child continuing to be febrile over the last 12 hours satisfied the “suspected typhoid fever” criteria and blood culture was performed. After a blood culture report positive for typhoid fever, a study physician assessed the child and treated him/her with oral azithromycin or referred the child to a higher center if the episode warranted hospitalization.

For shedding, a child with blood culture-confirmed typhoid fever was designated as an “index case” in his/her household and a written informed consent was obtained for the household’s participation. A “secondary case” was defined as a household member/contact of the index case and was subsequently identified with blood culture-confirmed typhoid fever. In households with index cases, a detailed history of fever-related illness and treatment among household members/contacts was collected for a period of 3 weeks prior to the onset of typhoid fever in the index child.

Given the intermittent shedding of *S* Typhi, the child was requested to provide 3 samples a week on alternate days, over the next 3 weeks for a total of 9 samples. The household members/contacts were also requested to provide 3 stool samples each, on alternate days over the next week. Data on the baseline as well as clinical characteristics for the typhoid fever episode were extracted from the database.

“Acute shedding” was defined as *S* Typhi shedding from the date of onset of illness to 1 month after the completion of antimicrobial therapy, whereas “convalescent shedding” was shedding between 1 and 12 months after antimicrobial therapy [15]. The typhoid cases/household members or contacts who shed *S* Typhi were followed up until 2 consecutive stool samples were negative for *S* Typhi. Stool samples were tested using quantitative real-time polymerase chain reaction (qPCR) at the Wellcome Trust Research Laboratory, Christian Medical College, Vellore, India.

The study protocol was approved by the Institutional Review Board (IRB) of Christian Medical College, Vellore, India (IRB Minute No.: 11867).

### Laboratory Methods

Stool samples collected were transported within 1–2 hours of collection at 4°C in vaccine carriers to the Wellcome Trust Research Laboratory. At the laboratory, samples were aliquoted and stored at −70°C. Before testing, stool samples were thawed, followed by deoxyribonucleic acid (DNA) extraction from a 20% stool suspension (W/V) using QIAamp Fast DNA stool mini kit (catalog no. 51604; QIAGEN, Hilden, Germany). The extracted DNA was used as a template in a real-time PCR (qPCR) assay for detection of the STY0201 gene of *S* Typhi [16]. Primers and probe sequences used were as follows: forward primer (5’-CGCGAAGTCAGAGTCGACATAG-3’), reverse primer (5’-AAGACCTCAACGCCGATCAC-3’), and probe (5’-FAM-CATTTGTTCTGGAGCAGGCTGACGG-BHQ1-3’) [16]. Five microliters of template DNA and 20 μL of qPCR master mixes were used for the amplification, and all of the qPCR assays were cycled under the following conditions: 50°C for 2 minutes, 95°C for 15 minutes, followed by 40 cycles at 95°C for 30 seconds, 60°C for 30 seconds, and 72°C for 30 seconds. Known positive and negative controls were included in each assay. A threshold cycle (Ct) value of ≤35 was the cutoff for positivity.

### Statistical Analysis

*Salmonella* Typhi shedding in untreated cases of typhoid fever was estimated to be ~10% based on stool culture. Because the SEFI study children were initiated on antimicrobials when culture results were obtained, the lower limit of precision estimates was expected to be 0%. Thus, the sample size was calculated using the formula 95% confidence interval (CI), [0, 3/n], where 100 cases were required to yield a precision range between 0% and 3% [17]. Because the study found a high rate of *S* Typhi shedding in the typhoid cases than expected, enrollment was stopped at 70 children with blood culture-confirmed typhoid fever, including both index and secondary cases.

All enrolled children were included in the final analyses. Categorical variables including *S* Typhi shedders and non-shedders, households with a member(s)/contact(s) being shedders and non-shedders, and sociodemographic variables are presented as percentages. Median and interquartile range (IQR) represents the duration of *S* Typhi shedding from the date of onset of fever in typhoid cases, duration of fever, duration of antibiotic usage, and the highest temperature recorded during the episode. Proportions across the groups were compared using the χ ^2^ test and in the case of continuous variables, *t* test or Mann-Whitney *U* test were used, based on normality of the data. Statistical significance was set at *P* < .05. Association between clinical characteristics and presence of shedding in typhoid cases were presented using odds ratios (ORs) with 95% CI.

## RESULTS

### Shedding in Index Cases

Between November 2018 and September 2019, 78 blood culture-confirmed typhoid fever cases from 67 households were detected in the Vellore SEFI cohort (Table 1). Seventy of the 78 (89.7%) typhoid fever cases from 63 of the 67 (94%) households consented to participation in the shedding substudy. Among 70 typhoid cases, 63 presented as index cases and 7 as secondary cases in study households. Characteristics of sample collection, shedding pattern, and associated clinical characteristics during typhoid fever are described for all 70 cases together. Eighteen of the 70 (25.7%) typhoid cases were hospitalized during their illness.

**Table 1.** Overview of the Children With Blood-Culture Confirmed Typhoid Fever (From SEFI Cohort) Enrolled in the Study on Stool Carriage

Variable	n (%)
Number of children with blood culture-confirmed typhoid fever in the SEFI cohort between November 2018 and June 2019	78
Number of children with blood culture-confirmed typhoid fever who consented to participate in the stool carriage study	70^a^/78 (89.7)
Number of children with typhoid fever who shed *Salmonella* Typhi in stool	16^b^/70 (22.9)
Number of children with typhoid fever who shed *S* Typhi in stool after the onset of fever	15/70 (21.4)
Median time (IQR) to shed *S* Typhi in stool from blood culture positivity, days	14 (8–21)
Number of children with stool samples positive for *S* Typhi more than once in the follow-up period	3/16 (18.8)

Abbreviations: IQR, interquartile range; SEFI, Surveillance for Enteric Fever in India.

^a^These 70 children resided within 63 households.

^b^One child was under follow-up as a household contact initially when his sibling was the index case for the particular household. Subsequently, this child developed fever and was also found to be typhoid positive by blood culture. Hence, we were able to find out that this child shed *S* Typhi 4 days before the confirmation of typhoid fever by blood culture, thereby having been an incubatory carrier.

Stool sample collection (first sample for the index case) began in the second week of illness, with a median time of 13 (IQR, 10–16) days, because the final blood culture report took several days. In children who were hospitalized, stool sample collection was initiated after discharge. The median number of stool samples per typhoid case was 9 (IQR, 8–9). A total of 557 stools were collected from the 70 children and 19 (3.4%) samples had *S* Typhi. The median Ct value in PCR was 34.6 (range, 26.7–35) (data not presented).

**Figure 1.**
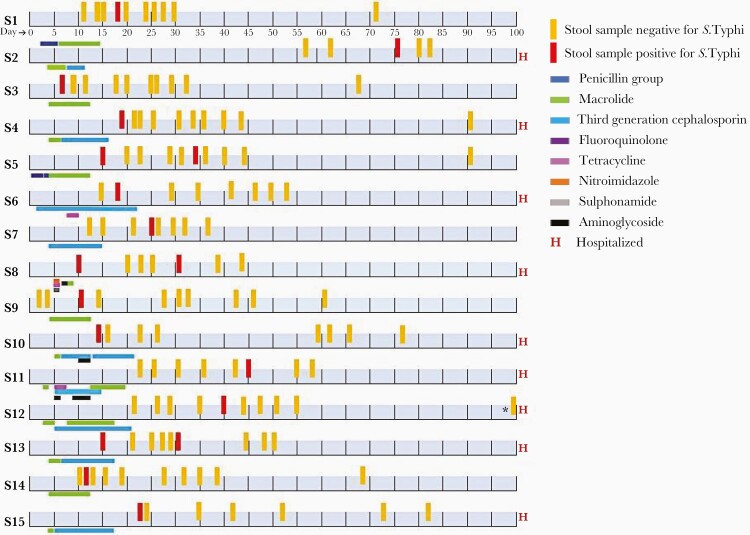
Time points of stool sample collection, *Salmonella* Typhi positivity in stool (*S* Typhi stool shedder), and antibiotic usage in the 15 (1 child who shed S Typhi prior to the onset of fever was not included in the figure) index children (S1–S15) followed up after blood culture positivity for *S* Typhi. *Stool sample collected on day 112.

Fourteen children shed *S* Typhi in stool at a median of 14 days after positive blood culture. One child had a sibling who was blood culture positive, shed *S* Typhi, and then became a case confirmed by blood culture. Another child shed bacteria 76 days after blood culture, during the convalescent phase. Three children had 2 samples each that were positive at different time-points (Figure 1).

The baseline characteristics of children who excreted *S* Typhi (n = 15) or not (n = 55) were comparable (Table 2). The mean maximal temperature recorded during fever episodes did not differ significantly between children who shed *S* Typhi and those who did not (103°F [standard deviation {SD}, 1.1] versus 102.8°F [SD, 1.3], *P* = .883) (Table 3). There was no significant difference in diarrhea (5 [33.3%] versus 11 [20%], *P* = .276) or abdominal pain (9 [60%] versus 24 [43.6%], *P* = .260) between S Typhi shedders and non-shedders. Children with culture-confirmed typhoid fever who shed S Typhi were 7 times more likely to be hospitalized when compared to the non-shedders (OR, 7.7; 95% CI, 1.8–32.6). Ten of 15 children shed *S* Typhi after the completion of the antibiotic course at a median of 11.5 (range, 3–61) days from the end of antibiotic therapy. The median duration of antibiotic usage was higher in shedders compared with nonshedders (13 [IQR, 12–17] versus 11 [IQR, 12–13], *P* = .05). However, shedders had fewer median days of treatment with azithromycin compared with nonshedders (9 [IQR, 2–10] versus 10 [IQR, 10–11], *P* = .003), because children who were hospitalized received intravenous antibiotics instead of oral azithromycin (Table 3). Azithromycin was the mainstay of treatment for 52 of the 70 typhoid fever cases, who presented as outpatients. Among 18 of the 70 children who were hospitalized during typhoid fever episode, 16 received intravenous third-generation cephalosporins and 2 received intravenous gentamycin, in addition to oral antibiotics (data not presented).

**Table 2.** Baseline Characteristics of Children Enrolled in the Stool Carriage Study Categorized as Shedders and Nonshedders of *Salmonella* Typhi (N = 70)

Baseline characteristics	S Typhi Shedder (n = 15 ^a^)	S Typhi Nonshedder (n = 55)	P Value
Age, median (IQR), years	8.4 (5.4–10.1)	7.8 (5.8–10.7)	.780
Sex			
Male	10 (66.7)	31 (56.4)	.473
Female	5 (33.3)	24 (43.6)	
Religion			
Hindu	11 (73.3)	31 (56.4)	.465
Christian	1 (6.7)	4 (7.3)	
Muslim	3 (20)	20 (36.4)	
Socioeconomic Status^b^			
Low	9 (60)	38 (69.1)	.506
Middle	6 (40)	17 (30.9)	
Source of Drinking Water^c^			
Unimproved (municipal water)	14 (93.3)	51 (92.7)	.936
Improved (bore well/commercially packaged)	1 (6.7)	4 (7.3)	
Toilet Facility^d^			
Unimproved sanitation	13 (86.7)	47 (85.5)	.905
Improved sanitation	2 (13.3)	8 (14.6)	
Presence of Crowding^e^			
Yes	4 (26.7)	13 (23.6)	.808
No	11 (73.3)	42 (76.4)	

Abbreviations: IQR, interquartile range.

^a^The one child who was an incubatory carrier, with subsequent stool samples being negative after blood culture positivity was not included as an *S* Typhi shedder.

^b^Socioeconomic status classification was done using modified Kuppusamy scale that accounted for occupation, education, and selected assets [19].

^c^Municipal water supply was classified as an unimproved drinking water because of it being at-risk of contamination before the point of collection at the household level (drinking water samples were tested regularly as a part of the SEFI protocol with data published elsewhere) [20].

^d^Households with toilets with its effluents being discharged into drains without treatment were classified to have “unimproved” sanitation, and those households with effluents from toilets being discharged into septic tanks were classified to have “improved” sanitation.

^e^Households that had more than 4 persons dwelling per room were classified as those with “crowding.”

**Table 3.** Clinical Characteristics of *Salmonella* Typhi Stool Shedders and Nonshedders (N = 70)

Clinical Characteristic	S Typhi Shedder	Nonshedder	P Value	OR (95% CI)
	(n = 15) (%)	(n = 55) (%)		
Fever				
Median (IQR) duration of fever, days	8 (7–11)	8 (7–10)	.883	-
Mean (SD) temperature in the fever episode, °F	103.1 (1.1)	102.8 (1.3)	.384	-
Vomiting present	7 (46.7)	24 (43.6)	.834	1.1 (0.3–4.1)
Diarrhea present	5 (33.3)	11 (20)	.276	2 (0.4–8.1)
Abdominal pain present	9 (60)	24 (43.6)	.260	1.9 (0.5–7.5)
Jaundice present	1 (6.7)	1 (1.8)	.318	3.9 (0.1–308.2)
Antibiotic Usage During the Typhoid Fever Episode				
Time of initiation of antibiotic from the onset of fever, days, median (IQR)	3 (2–3)	3 (3–4)	.131	-
Total duration of antibiotic usage, days, median (IQR)	13 (12–17)	11 (10–13)	.05	-
Duration of azithromycin^a^ usage, days, median (IQR)	9 (2–10)	10 (10–11)	**.003**	
Hospitalization Details				
Children hospitalized for the typhoid fever episode	9 (60)	9 (16.4)	**<.001**	**7.7 (1.8–32.6)**
Duration of hospitalization, days, median (IQR) (n = 9)	6 (5–8)	7 (5–7)	.894	-
Antibiotic Susceptibility Testing for Ciprofloxacin^b^				
Complete sensitivity	0 (0)	1 (1.8)	.870	-
Intermediate sensitivity	13 (86.7)	47 (85.5)		
Complete resistance	2 (13.3)	7 (12.7)		

P < .05 is statistically significant and is presented in bold text.

Abbreviations: CI, confidence interval; IQR, interquartile range; OR, odds ratio; SD, standard deviation.

^a^Azithromycin was given to all children with blood culture-positive typhoid fever as per the study protocol.

^b^All of the 70 *S* Typhi strains isolated by blood culture were completely sensitive to ampicillin, chloramphenicol, co-trimoxazole, azithromycin, and ceftriaxone.

### Shedding in Household Contacts

Fifty-six of the 63 households with index typhoid cases provided stool samples from at least 1 member. The median number of household contacts who provided stool samples was 3 (IQR, 2–4). The median time to the first stool sample from household contacts was 13 (IQR, 10–15.5) days, from the onset of typhoid fever in the index case. A total of 487 stool samples were collected from 172 household members/contacts and the *S* Typhi PCR positivity rate was 2.9% (14 of 487), with median Ct values of 34.4 (range, 30.1–34.9). Seven of 56 households reported secondary cases of blood culture-confirmed typhoid fever, with the median serial interval for the onset of fever between secondary and index cases in these households being 12 (IQR, 6–16) days (data not presented).

Overall, 12 of the 172 (7%) household contacts in 11 of the 56 (19.6%) households shed *S* Typhi. Of the 11 households, one particular household had two individuals who were infected and shed S Typhi (Table 4). Of the 12 contacts with shedding, 5 were asymptomatic, whereas 7 had a fever, 4 of whom were subsequently confirmed by blood culture as typhoid. The median age of symptomatic household members/contacts who shed *S* Typhi was lower than that of asymptomatic members/contacts with shedding [5 (2–15) years versus 35 (32–42) years] (Table 5). Ten of the 12 household contacts provided repeat stool samples 2 months from the time of stool positivity, and all tested negative.

**Table 4.** Presence of Concurrent Stool Shedders of *Salmonella* Typhi Within the 56 Households of Typhoid-Positive Children

Variable	N (%)
Number of households with index case that had at least 1 family member shedding *S* Typhi in stool	11^a^/56 (19.6%)
Number of household contacts (n = 172) from 56 households who were found to shed *S* Typhi in stool	12/172 (7%)
Number of contacts who shed *S* Typhi more than once during follow up	2/12 (16.7%)

^a^Four and 7 households had *S* Typhi shedders who were asymptomatic and symptomatic, respectively. Of the 7 households with symptomatic shedders, 3 had a shedder and each had history of fever; the other 4 households had sibling children as shedders and typhoid fever had been confirmed by blood culture.

**Table 5.** Characteristics of the Household Contacts Within the 11 Households of Typhoid Positive Children^a^ Who Shed *Salmonella* Typhi in Stool (n = 12)

Household ID	Age of the Household Contact, Years	Sex	Relationship	No. of Positive Household Contacts	Symptomatic/Asymptomatic	Time to Positivity From Fever Onset in Symptomatic Contacts, Days
H1	15	F	Sister	1	Symptomatic	18
H2	1	F	Sister	1	Symptomatic	23
H3	27	F	Mother	1	Symptomatic	11
H4	5	M	Brother	1	Symptomatic^a^	−3^b^
H5	9	F	Sister	1	Symptomatic^a^	6
H6	4	F	Sister	1	Symptomatic^a^	10
H7	2	F	Sister	1	Symptomatic^a^	18
H8	42	F	Mother	1	Asymptomatic	-
H9	65	M	Grandfather	2	Asymptomatic	-
	32	F	Mother		Asymptomatic	-
H10	35	F	Mother	1	Asymptomatic	-
H11	8	M	Brother	1	Asymptomatic	-

^a^Blood culture-confirmed typhoid fever.

^b^This child, a sibling of an index case, shed *S* Typhi 3 days before the onset of typhoid fever as a result of testing of stool sample that was collected for all household members of the index case.

## Discussion

This study is the first attempt from an Indian setting to use a longitudinal approach in a cohort of children with blood culture-confirmed typhoid fever to estimate *S* Typhi shedding in stool and to further understand household transmission. We found that approximately 1 in 5 children with blood culture-confirmed typhoid fever shed *S* Typhi in stool. This shedding was notable in the first month of illness, followed by a cessation in shedding thereafter. This pattern of secondary shedding in typhoid cases has been documented previously in other hospital-based studies [10, 12, 18]. This finding from our cohort holds important relevance given that this pattern of shedding could potentially drive the transmissibility of *S* Typhi from the case(s) to contacts, especially children, at households as well as schools. Studies that have described dynamics of *S* Typhi shedding previously were predominantly among the hospitalized typhoid cases, in contrast to our study where children from a cohort under active surveillance for fever were followed up in a community setting, making the case for *S* Typhi transmission between shedders and contacts stronger [10, 18].

 The role of antibiotics in relation to *S* Typhi shedding is an important aspect investigated in this cohort. It is important to note that 14% of children with typhoid in our study shed *S* Typhi even after the completion of adequate antimicrobial therapy. Previous studies based on stool culture have documented a remarkable reduction in bacillary shedding from 27% to <6%, after treatment with azithromycin [21–27]. Although azithromycin was shown to be effective in clearing *S* Typhi in these studies, we noted a post-antibiotic shedding in our study children of up to 14%. This could probably be attributed to the use of highly sensitive PCR in our study when compared with stool cultures used in other studies. Furthermore, the possibility of persistence of *S* Typhi in the gut with an altered intestinal microbiota as a result of antibiotic therapy, which is specific to the children in this setting, cannot be ruled out and needs further exploration [9, 28].

Children hospitalized for typhoid fever in our study had 7.7 times higher odds of shedding *S* Typhi. To understand this association, we explored the clinical severity of typhoid among shedders who were hospitalized, compared with those not hospitalized. Hospitalized children reported fever of high grade and prolonged duration, vomiting, and diarrhea at significantly higher rates compared to those treated as outpatients for typhoid fever (data not shown). Consistent with our findings, a hospital-based study from Vietnam reported a higher *S* Typhi shedding of 27% in patients who concurrently reported gastrointestinal symptoms at a higher proportion [21]. Therefore, *S* Typhi shedding in stool is perhaps a function of the clinical severity in patients with typhoid fever. Furthermore, hospitalized typhoid cases in our study received antibiotics for a longer duration compared to those not hospitalized (17 and 11 days, respectively). This prolonged course of antibiotics in these hospitalized children perhaps leads to an intestinal dysbiosis, with an upregulated *S* Typhi persistence in the gut, and subsequently shedding, and this has been documented with *Salmonella* spp in humans as well as mice models [29].

It is worth noting that this study captured information on *S* Typhi shedding among household members/contacts of the index cases. Approximately one fifth of the households with typhoid-positive children had at least 1 household member/contact shedding *S* Typhi. Approximately 40% of these household member/contacts who were shedders were asymptomatic and mostly adults. This is an important finding because adults, although clinically immune to typhoid given the repeated exposures in endemic settings, could potentially add to *S* Typhi transmission as asymptomatic shedders in households. Furthermore, a comparison of the time of onset of fever in symptomatic shedders with the index cases in the respective households showed that the fever onset dates within each household fell within a single time frame of 2 weeks. Although this points towards a recent *S* Typhi infection within the same household, further ascertainment of transmission from a common point source such as contaminated drinking water or a household contact is challenging, and this is beyond the scope of the study.

Controlled human infection models have shown that the Typhoid conjugate vaccine (TCV) decreases *S* Typhi shedding in those exposed to the infection [12]. Improving water and sanitation along with TCV introduction is pivotal in developing settings like India to reduce transmission and the disease burden. The strengths of this study are the intensive and systematic follow up of blood culture-confirmed typhoid-positive children and the incorporation of the highly sensitive qPCR to detect stool shedding of *S* Typhi in place of stool culture (sensitivity <40%) [22, 23]. This study has a few limitations. The study could not capture *S* Typhi shedding during the incubatory and early phase of illness, given that shedding is highest during the early part of typhoid fever [12]. Because stool samples were collected in the second week of illness or after children were discharged from the hospital, episodes of shedding may have been missed during the acute phase of infection. In addition, prior studies have used stool culture to determine shedding; thus, it is difficult to make direct comparisons between this study and prior studies. Furthermore, ascertaining household transmission based on a positive PCR is unclear, because it may pick up both live and dead organisms in the test.

## Conclusions

In conclusion, our study documents a high proportion of children under 15 years of age shedding *S* Typhi, given that children constitute a major bulk of the typhoid disease burden in endemic settings. *Salmonella* Typhi shedding was observed even with the timely initiation of antimicrobial therapy. These children recovering from typhoid fever could be potential drivers of typhoid disease transmission in communities with suboptimal water and sanitation. The detection and treatment of typhoid carriers is challenging. However, typhoid control activities such as the national introduction of Typhoid conjugate vaccine, in concurrence with efforts to strengthen water and sanitation, is pivotal to break the transmission cycle of *S* Typhi in high-burden settings.

## Notes

***Acknowledgments.*** We thank the parents and the children of the Surveillance for Enteric Fever in India (SEFI) study. We also thank Nandhini M and Annai Gunasekaran who supervised the field activities and the SEFI field research assistants who were involved in the sample and data collection.

***Author contributions.*** G. K., N. C. G., V. R. M., and M. S. conceived the study, drafted the original protocol, and provided critical revision of the final draft. M. S., K. N. S., and S. G. were involved in the conduct of the study, performed statistical analyses, and wrote and revised the manuscript. S. G. and N. K. were in charge of the laboratory assays. All authors approved the final version of the manuscript; they agree to be accountable for all aspects of the work in ensuring that questions related to the accuracy or integrity of any part of the work are appropriately investigated and resolved; and they confirm that all persons designated as authors qualify for authorship and all those who qualify for authorship are listed.

***Supplement sponsorship.*** This supplement is sponsored by the Christian Medical College Vellore Association.

***Financial support.*** The study was funded by a grant from the Bill & Melinda Gates Foundation (OPP1159351; to G. K.).

***Potential conflicts of interest.*** All authors: No reported conflicts of interest. All authors have submitted the ICMJE Form for Disclosure of Potential Conflicts of Interest.

## References

[1] Stanaway JD , Reiner RC, Blacker BF, et al. The global burden of typhoid and paratyphoid fevers: a systematic analysis for the global burden of disease study 2017. Lancet Infect Dis 2019; 19:369–81. 30792131 10.1016/S1473-3099(18)30685-6 PMC6437314

[2] John J , Van Aart CJ, Grassly NC. The burden of typhoid and paratyphoid in India: systematic review and meta-analysis. PLoS Negl Trop Dis 2016; 10:e0004616. 27082958 10.1371/journal.pntd.0004616 PMC4833325

[3] Mogasale VV , Ramani E, Mogasale V, Park JY, Wierzba TF. Estimating typhoid fever risk associated with lack of access to safe water: a systematic literature review. J Environ Public Health 2018; 2018:9589208. 30174699 10.1155/2018/9589208 PMC6076975

[4] Luby SP . Urban slums: a supportive ecosystem for typhoidal salmonellae. J Infect Dis 2018; 218:250–4. 10.1093/infdis/jiy324 PMC6226803 30060082

[5] Crump JA . Progress in typhoid fever epidemiology. Clin Infect Dis 2019; 68:4–9. 10.1093/cid/ciy846 PMC6376096 30767000

[6] Akullian A , Ng’eno E, Matheson AI, et al. Environmental transmission of typhoid fever in an urban slum. PLoS Negl Trop Dis 2015; 9:e0004212. 26633656 10.1371/journal.pntd.0004212 PMC4669139

[7] Krzyzanowski F Jr , Zappelini L, Martone-Rocha S, et al. Quantification and characterization of *Salmonella* spp. isolates in sewage sludge with potential usage in agriculture. BMC Microbiol 2014; 14:263. 25927729 10.1186/s12866-014-0263-x PMC4207900

[8] Sahlström L , Jong BD, Aspan A. Salmonella isolated in sewage sludge traced back to human cases of salmonellosis. Lett Appl Microbiol 2006; 43:46–52. 16834720 10.1111/j.1472-765X.2006.01911.x

[9] Monack DM . Salmonella persistence and transmission strategies. Curr Opin Microbiol 2012; 15:100–7. 22137596 10.1016/j.mib.2011.10.013

[10] Parry CM , Hien TT, Dougan G, White NJ, Farrar JJ. Typhoid fever. N Engl J Med 2002; 347:1770–82. 12456854 10.1056/NEJMra020201

[11] Hornick RB , Greisman SE, Woodward TE, DuPont HL, Dawkins AT, Snyder MJ. Typhoid fever: pathogenesis and immunologic control. N Engl J Med 1970; 283:686–91. 4916913 10.1056/NEJM197009242831306

[12] Gibani MM , Voysey M, Jin C, et al. The impact of vaccination and prior exposure on stool shedding of *Salmonella typhi* and *Salmonella paratyphi* in 6 controlled human infection studies. Clin Infect Dis 2019; 68:1265–73. 30252031 10.1093/cid/ciy670 PMC6452003

[13] World Health Organization. Typhoid vaccines: WHO position paper , March 2018. Week Epidemiol Rec 2018; 93:153–72. 10.1016/j.vaccine.2018.04.022 29661581

[14] John J , Bavdekar A, Rongsen-Chandola T, Dutta S, Kang G; NSSEFI Collaborators. Estimating the incidence of enteric fever in children in India: a multi-site, active fever surveillance of pediatric cohorts. BMC Public Health 2018; 18:594. 29724223 10.1186/s12889-018-5498-2 PMC5934828

[15] World Health Organization. Typhoid and other invasive salmonellosis - surveillance standards. Available at: https://www.who.int/immunization/monitoring_surveillance/burden/vpd/WHO_SurveillanceVaccinePreventable_21_Typhoid_BW_R1.pdf?ua=1. Accessed 18 May 2020.

[16] Nga TV , Karkey A, Dongol S, et al. The sensitivity of real-time PCR amplification targeting invasive *Salmonella* serovars in biological specimens. BMC Infect Dis 2010; 10:125. 20492644 10.1186/1471-2334-10-125 PMC2886058

[17] Selvin S. Statistical Power and Sample Size Calculations. In: Statistical Analysis of Epidemiologic Data (Monographs in Epidemiology and Biostatistics). 3rd ed. New York: Oxford University Press; 2004; 75–87.

[18] Gilman RH , Terminel M, Levine MM, Hernandez-Mendoza P, Hornick RB. Relative efficacy of blood, urine, rectal swab, bone-marrow, and rose-spot cultures for recovery of *Salmonella* typhi in typhoid fever. Lancet 1975; 1:1211–3. 48834 10.1016/s0140-6736(75)92194-7

[19] Kattula D , Venugopal S, Velusamy V, et al. Measuring poverty in Southern India: a comparison of socio-economic scales evaluated against childhood stunting. PLoS One 2016; 11:e0160706. 10.1371/journal.pone.0160706 PMC4973914 27490200

[20] Srinivasan M , Sindhu KN, Kumar SJ, et al. Hepatitis A outbreak with the concurrence of *Salmonella* Typhi and *Salmonella* Poona infection in children of urban Vellore, South India. Am J Trop Med Hyg 2020; 102:1249–52. 32228778 10.4269/ajtmh.19-0742 PMC7253137

[21] Parry CM , Ho VA, Phuong le T, et al. Randomized controlled comparison of ofloxacin, azithromycin, and an ofloxacin-azithromycin combination for treatment of multidrug-resistant and nalidixic acid-resistant typhoid fever. Antimicrob Agents Chemother 2007; 51:819–25. 17145784 10.1128/AAC.00447-06 PMC1803150

[22] Chinh NT , Parry CM, Ly NT, et al. A randomized controlled comparison of azithromycin and ofloxacin for treatment of multidrug-resistant or nalidixic acid-resistant enteric fever. Antimicrob Agents Chemother 2000; 44:1855–9. 10858343 10.1128/aac.44.7.1855-1859.2000 PMC89974

[23] Butler T , Sridhar CB, Daga MK, et al. Treatment of typhoid fever with azithromycin versus chloramphenicol in a randomized multicentre trial in India. J Antimicrob Chemother 1999; 44:243–50. 10473232 10.1093/jac/44.2.243

[24] Dolecek C , Tran TP, Nguyen NR, et al. A multi-center randomised controlled trial of gatifloxacin versus azithromycin for the treatment of uncomplicated typhoid fever in children and adults in Vietnam. PLoS One 2008; 3:e2188. 18493312 10.1371/journal.pone.0002188 PMC2374894

[25] Frenck RW Jr , Nakhla I, Sultan Y, et al. Azithromycin versus ceftriaxone for the treatment of uncomplicated typhoid fever in children. Clin Infect Dis 2000; 31:1134–8. 11073741 10.1086/317450

[26] Frenck RW Jr , Mansour A, Nakhla I, et al. Short-course azithromycin for the treatment of uncomplicated typhoid fever in children and adolescents. Clin Infect Dis 2004; 38:951–7. 15034826 10.1086/382359

[27] Girgis NI , Butler T, Frenck RW, et al. Azithromycin versus ciprofloxacin for treatment of uncomplicated typhoid fever in a randomized trial in Egypt that included patients with multidrug resistance. Antimicrob Agents Chemother 1999; 43:1441–4. 10348767 10.1128/aac.43.6.1441 PMC89293

[28] Gopinath S , Carden S, Monack D. Shedding light on *Salmonella* carriers. Trends Microbiol 2012; 20:320–7. 22591832 10.1016/j.tim.2012.04.004

[29] Gal-Mor O . Persistent infection and long-term carriage of typhoidal and nontyphoidal salmonellae. Clin Microbiol Rev 2018; 32:e00088–18. 30487167 10.1128/CMR.00088-18 PMC6302356

